# Annual acknowledgement of BMC Medicine manuscript reviewers

**DOI:** 10.1186/s12916-016-0569-7

**Published:** 2016-01-29

**Authors:** Sabina Alam

**Affiliations:** BioMed Central Ltd, 236 Gray’s Inn Road, London, WC1X 8HB UK

## Abstract

The editors of *BMC Medicine* would like to thank all our reviewers who have contributed to the journal in Volume 13 (2015).

**John Aaskov**

Australia

**Rebecca Abbott**

Australia

**Nicole Achee**

USA

**Zanfina Ademi**

Switzerland

**Alessio Aghemo**

Italy

**Kingsley Agho**

Australia

**Joseph Ahearn**

USA

**Golo Ahlenstiel**

Australia

**Mohamed Azmi Ahmad Hassali**

Malaysia

**Marco Ajelli**

Italy

**Emilio Alba**

Spain

**Urs-Vito Albrecht**

Germany

**Mohammad Ali**

USA

**Marjan Alssema**

Netherlands

**Isabel Amendoeira**

Portugal

**Annie Anderson**

UK

**Christina Annunziata**

USA

**Alessandro Antonelli**

Italy

**Katerina Antoniou**

Greece

**Andrew Armstrong**

USA

**David Armstrong**

USA

**Michael Ashton**

Sweden

**Vasilios Athyros**

Greece

**Titus Augustine**

UK

**Dagfinn Aune**

UK

**Kari Auranen**

Finland

**Philippe Autier**

France

**Anthony Avery**

UK

**Angelo Avogaro**

Italy

**Makoto AYABE**

Japan

**Jose Luis Ayuso-Mateos**

Spain

**Maciej Banach**

Poland

**Jesus Banales**

Spain

**Amitava Banerjee**

UK

**Emily Banks**

Australia

**Gustavo Barja**

Spain

**Quique Bassat**

Spain

**Imelda Bates**

UK

**Michael Bauer**

Germany

**Harry Bear**

USA

**James Beeson**

Australia

**Caleb Behrend**

USA

**Philip Bejon**

UK

**Francois Beland**

Canada

**Laurent Belec**

France

**Himisha Beltran**

USA

**Merav Ben Natan**

Israel

**Jacob Berlin**

USA

**Robert Berry**

USA

**Abbas Bhuiya**

Bangladesh

**Zulfiqar Bhutta**

Canada

**Fausto Biancari**

Italy

**Peter Biberthaler**

Germany

**Godfrey Biemba**

Zambia

**Leslie Biesecker**

USA

**Colin Binns**

Australia

**Giuseppe Biondi-Zoccai**

Italy

**Carl Birmingham**

Canada

**Peter Bitterman**

USA

**Alan Bittles**

Australia

**Giovanni Blandino**

Italy

**Josè Enrique O'Connor Blasco**

Spain

**Sally Blower**

USA

**Stefanos Bonovas**

Greece

**Marilyn Bookbinder**

USA

**Matthias Borchert**

Germany

**Soo Borson**

USA

**Patrick Bossuyt**

Netherlands

**Emmanuel Bottieau**

Belgium

**Teun Bousema**

UK

**Isabelle Boutron**

France

**Anne Marie Bouvier**

France

**John Bowman**

USA

**Katharina Braun**

Germany

**George Bray**

USA

**Tara Breslin**

USA

**Alexandros Briasoulis**

USA

**Andre Briend**

France

**Richard Brilli**

USA

**Louise Brinton**

USA

**Thomas Brix**

Denmark

**Marion Broome**

USA

**Wolfgang Brück**

Germany

**Martin Bruene**

Germany

**Chris Bullen**

New Zealand

**Linda Bullock**

USA

**Frances Bunn**

UK

**Pierre-Regis Burgel**

France

**Jeremy Burton**

Canada

**Philip Butcher**

UK

**Monique Cadrin**

Canada

**Carlos Caldas**

UK

**Valentina Cambiano**

UK

**Ian Cameron**

Australia

**Benjamin Campbell**

USA

**Diana Campioni**

Italy

**Havi Carel**

UK

**Elisabetta Cariani**

Italy

**Waldemar Carlo**

USA

**Waldemar Carlo**

USA

**Edgardo D. Carosella**

France

**Jason Carroll**

UK

**Joan Cayla**

Spain

**Guillaume Chabot-Couture**

USA

**Marc Chamberlain**

USA

**Leslie Chan**

Canada

**Larry Chang**

USA

**Helena Chang**

USA

**Saurav Chatterjee**

USA

**Cynthia Chee**

Singapore

**Francis Chinegwundoh**

UK

**Yen-Hung Chow**

Taiwan

**Gerardo Chowell**

USA

**Renata Cifkova**

Czech Republic

**Andrea Cipriani**

UK

**Daniela Cirillo**

Italy

**Michael Clark**

UK

**Nicholas Clement**

UK

**Stephen Cochi**

USA

**Ted Cohen**

USA

**Jan Willem Cohen Tervaert**

Netherlands

**Vittoria Colizza**

France

**Clare Collins**

Australia

**Paul Colson**

USA

**David Conen**

Switzerland

**Cristophe Corpechot**

France

**Alberto Corsini**

Italy

**Alister Craig**

UK

**Bart Criel**

Belgium

**Miroslava Čuperlović-Culf**

Canada

**Harel Dahari**

USA

**Synneve Dahlin-Ivanoff**

Sweden

**Ingrid Dahlman**

Sweden

**Mary D'Alton**

USA

**Teresa Damush**

USA

**Janet Dancey**

Canada

**Lalit Dandona**

India

**Carrie Daniel MacDougall**

USA

**Anna-Karin Danielsson**

Sweden

**Fabrizio D'ascenzo**

Italy

**Paban Kumar Dash**

India

**Adil Daud**

USA

**Ayesha De Costa**

Sweden

**Jan De Lepeleire**

Belgium

**Michel de Lorgeril**

France

**Paola De Rango**

Italy

**Luc de Witte**

Netherlands

**Matthew DeCamp**

USA

**Agnes Dechartres**

France

**Martin Dedicoat**

UK

**Justin T Denholm**

Australia

**Ralph Depalma**

USA

**Rumona Dickson**

UK

**Anna Diehl**

USA

**Roland Diel**

Germany

**Peter John Diggle**

UK

**Mary Dixon-Woods**

UK

**Helen Dolk**

UK

**Joseph Donnelly**

USA

**Susan Dorman**

USA

**David Dowdy**

USA

**Stephen Duffy**

UK

**Christine Durand**

USA

**Helena Earl**

UK

**Michael Engelgau**

USA

**Mike English**

Kenya

**Håkan Eriksson**

Sweden

**Markus Eszlinger**

Germany

**Arash Etemadi**

USA

**Jean-Francois Etter**

Switzerland

**Victoria Fan**

USA

**Rui Fang**

USA

**Yiru Fang**

China

**Laurent Fauchier**

France

**Seena Fazel**

UK

**Ian Fentiman**

UK

**Peter Ferenci**

Austria

**Cleusa Ferri**

Brazil

**Eduardo Ferriolli**

Brazil

**David Fidock**

USA

**Suzanne Filteau**

UK

**Lawrence Fisher**

USA

**Dale Fisher**

Singapore

**David Fisman**

Canada

**Adam Fleischer**

USA

**Kirsten Fleischmann**

USA

**Helen Fletcher**

UK

**Olivia Fletcher**

UK

**João Fonseca**

Portugal

**Theodoros Foukakis**

Sweden

**Anna Fracanzani**

Italy

**Kenneth Freedland**

USA

**Matthew Freiberg**

USA

**Vic Froelicher**

USA

**Rochelle Fu**

USA

**Jan Gaertner**

Germany

**Annemarie Gagnon**

Canada

**Emmanuela Gakidou**

USA

**Manoj Gambhir**

Australia

**Lourdes Garcia**

Mexico

**Michel Garenne**

France

**Amit Garg**

Canada

**Daniel Gaudet**

Canada

**Colin Geddes**

UK

**Karen Gelmon**

Canada

**Steven George**

USA

**Maureen George**

USA

**Ruth Gershoni-Baruch**

Israel

**Davina Ghersi**

Australia

**Balaram Ghosh**

India

**Peter Giannoudis**

UK

**John Gibson**

UK

**Massimo Girardis**

Italy

**Alan Girling**

UK

**John Gladman**

UK

**Martin Gleave**

Canada

**Brian Godman**

Sweden

**Su Golder**

UK

**Gabriela Gomes**

Portugal

**Guendalina Graffigna**

Italy

**Jonathan Grant**

UK

**Michael Gravett**

USA

**Darren C Greenwood**

UK

**Edward Gregg**

USA Minor Outlying Islands

**Ulla Griffiths**

UK

**Carlos Grilo**

USA

**Mitchell Gross**

USA

**Weihua Guan**

USA

**Jean-Claude Guédon**

Canada

**Miguel Gueimonde**

Spain

**Bruce Guthrie**

UK

**Balazs Györffy**

Hungary

**Carl Haasper**

Germany

**Peter Hajek**

UK

**Eelko Hak**

Netherlands

**Andrew Hall**

UK

**Philip Hannaford**

UK

**David Hansell**

UK

**Josh Hanson**

Australia

**Ubydul Haque**

USA

**Shamil Haroon**

UK

**Mark Harris**

Australia

**Elizabeth Hatch**

USA

**Phillipa Hay**

Australia

**Peiman Hematti**

USA

**Philip Hill**

New Zealand

**Marie-France Hivert**

Canada

**Allison Hodge**

Australia

**Michael Hoffmeister**

Germany

**Scott Holmberg**

USA

**Tim Holt**

UK

**Lotty Hooft**

Netherlands

**Robert Horsburgh**

USA

**Thomas House**

UK

**Allan House**

UK

**Christopher Howson**

USA

**Daniela Hozbor**

Argentina

**Richard Hubbard**

UK

**David Hui**

USA

**Carrie Huisingh**

USA

**Don Husereau**

Canada

**Lars Hviid**

Denmark

**Carel IJsselmuiden**

Switzerland

**Eiichi Ishikawa**

Japan

**Ken-ichi Ito**

Japan

**Till Ittermann**

Germany

**Richard Jabbour**

UK

**Hansen-Schwartz Jacob**

Denmark

**Hartmut Jaeschke**

USA

**Allan Jaffe**

USA

**Anders Jakobsen**

Denmark

**Prakash Mohan Jeena**

South Africa

**Gregor Jemec**

Denmark

**Prabhat Jha**

Canada

**Maria A Jimenez-Sousa**

Spain

**Martin Johansen**

Denmark

**Søren Paaske Johnsen**

Denmark

**Pedro Jose**

USA

**Andrew Jull**

New Zealand

**S. Patrick Kachur**

USA

**Louiza Kalokairinou**

Belgium

**Tatsuo Kanda**

Japan

**Pekka Kannus**

Finland

**Harin Karunajeewa**

Australia

**Monika Kastner**

Canada

**Pawel Kawalec**

Poland

**Mary Kawonga**

South Africa

**Kassoum Kayentao**

Mali

**Michael P Keane**

Ireland

**Richard Keefe**

USA

**William Keenan**

USA

**David Kelso**

USA

**Eben Kenah**

USA

**Gunter Kenis**

Netherlands

**Hector Keun**

UK

**Stefan Kiechl**

Austria

**Gerry Killeen**

Tanzania

**Hyung Kim**

USA

**Laurence Kirmayer**

Canada

**Niranjan Kissoon**

Canada

**Takanari Kitazono**

Japan

**Jos Kleijnen**

UK

**Kerstin Klipstein-Grobusch**

Netherlands

**Martin Kneyber**

Netherlands

**Gwenan Knight**

UK

**Martin Koebel**

Canada

**Amos Korczyn**

Israel

**Boris Kotchoubey**

Germany

**Anthony Kovac**

USA

**Christian Krauth**

Germany

**Mirjam Kretzschmar**

Netherlands

**Sanjeev Krishna**

UK

**Darcy Krueger**

USA

**Alexander Kulminski**

USA

**Fred Kummerow**

USA

**Ralph Kupka**

Netherlands

**Nathan Kuppermann**

USA

**Vitaly Kushnir**

USA

**Oliver Kuß**

Germany

**George Kyei**

USA

**Catherine Kyobutungi**

Kenya

**Dirk Lachenmeier**

Germany

**Sarah Lamb**

UK

**Edward Lammer**

USA

**Tom Lang**

USA

**Christine Laronga**

USA

**David Le Couteur**

Australia

**Peter Lee**

UK

**Brian Lehmann**

USA

**Sam Leinster**

UK

**Jean-Pascal Lemelin**

Canada

**Isabelle Leparc-Goffart**

France

**Kieron Leslie**

USA

**Justin Lessler**

USA

**Joel Lexchin**

Canada

**Adolfo Liao**

Brazil

**Evangelos Liberopoulos**

Greece

**Eric Lien**

USA

**Lina Lim**

Singapore

**Shu-Chun Lin**

Taiwan

**Benjamin Linas**

USA

**Marc Lipman**

UK

**Deborah Little**

USA

**Peter A. Littlejohns**

UK

**Li Liu**

USA

**Andrew Liu**

USA

**Yoon Kong Loke**

UK

**Chanthap Lon**

Thailand

**Joanne Lord**

UK

**Alison Loren**

USA

**Catherine Lozupone**

USA

**Giovanni Lughezzani**

Italy

**Xianghua Luo**

USA

**Alan Lyles**

USA

**Paul Lysaker**

USA

**Harriet MacMillan**

Canada

**Iain MacPhee**

UK

**Matthew Maddocks**

UK

**Nicola Maffulli**

UK

**Alan Magill**

USA

**Kathryn Maitland**

UK

**Marcin Majka**

Poland

**Ville-Petteri Makinen**

Australia

**Elizabeth Manias**

Australia

**Samuel Mann**

USA

**Joanne Mantell**

USA

**Douglas Manuel**

Canada

**Donatella Marazziti**

Italy

**Julia Marcus**

USA

**Paul Marik**

USA

**Donald Marion**

USA

**Maurie Markman**

USA

**Michael Marmura**

USA

**Louise Marston**

UK

**Miguel Martinez-Gonzalez**

Spain

**Wellington Martins**

Brazil

**Mauri J Marttunen**

Finland

**Ana Marusic**

Croatia

**Steven Marwaha**

UK

**Joseph Massaro**

USA

**Manjit Matharu**

UK

**Colin Mathers**

Switzerland

**Joseph Mathew**

India

**Dimitrios Matthaiou**

Greece

**Lawrence Mbuagbaw**

Canada

**Emma McBryde**

Australia

**Mary McCarron**

Ireland

**James McCarthy**

Australia

**Tim McHugh**

UK

**Martin McKee**

UK

**Ann-Marie McLaren**

Canada

**Candace Mcnaughton**

USA

**Elizabeth McQuaid**

USA

**Keith Meadows**

UK

**Didier Ménard**

Cambodia

**Stewart Mercer**

UK

**Tammy Meyers**

South Africa

**Saskia Middeldorp**

Netherlands

**David Miles**

UK

**Michael Miller**

USA

**Edward Mills**

Canada

**Carole Mitnick**

USA

**David Moher**

Canada

**Ben Mol**

Australia

**Marc Molendijk**

Netherlands

**Malcolm Edward Molyneux**

Malawi

**Wuelton Monteiro**

Brazil

**Filippo Montemurro**

Italy

**Ann Moormann**

USA

**Andrew Moran**

USA

**Larry Moreland**

USA

**Victor Moreno**

Spain

**John Morgan**

USA

**Margaret Morris**

Australia

**José María Mostaza**

Spain

**Shahrul Mt-Isa**

UK

**Zindoga Mukandavire**

UK

**Helen Murphy**

UK

**Paola Muti**

USA

**Kurt Naber**

Germany

**Sharon Nachman**

USA

**VasI Naganathan**

Australia

**Harish Nair**

UK

**Louise Nash**

Australia

**Florian Naudet**

France

**E Andrea Nelson**

UK

**Patrick Neven**

Belgium

**Anthony Newall**

Australia

**Mark Nicol**

South Africa

**Hiroshi Nishiura**

Japan

**Alessandro Nobili**

Italy

**Abdisalan Noor**

Kenya

**Ole Norheim**

Norway

**Alexis Nzila**

Saudi Arabia

**Jeremy Oats**

Australia

**Pat O'Campo**

Canada

**Daniel O'Connor**

Australia

**William Oh**

USA

**Kerstin M. Oltmanns**

Germany

**Eng Eong Ooi**

Singapore

**Walter Orenstein**

USA

**Ronan O'Toole**

Australia

**Jan Erik Otterstad**

Norway

**Hans Overgaard**

Norway

**Raj Padwal**

Canada

**Chi Pae**

North Korea

**Claire Palles**

UK

**Ankur Pandya**

USA

**Vassilios Papalois**

UK

**Carmine Pariante**

UK

**Jigisha Patel**

UK

**Andrew Patterson**

USA

**Friedemann Paul**

Germany

**Martha Payne**

USA

**Chris Pemberton**

New Zealand

**Guangyong Peng**

USA

**Robert Perlman**

USA

**Kerstin Persson Waye**

Sweden

**Jaime Peters**

UK

**Stefan Peterson**

Sweden

**Daniel Petrovic**

Slovenia

**Ronald Pfeiffer**

USA

**Siobhan Phillips**

USA

**Hilary Pinnock**

UK

**Mario Pirisi**

Italy

**Loris Pironi**

Italy

**Charlotta Pisinger**

Denmark

**Christopher Plowe**

USA

**Venerino Poletti**

Italy

**Riccardo Polosa**

Italy

**Phillippa Poole**

New Zealand

**Maarten Postma**

Netherlands

**Michael Postow**

USA

**Tatjana Potpara**

Serbia

**Peter Preiser**

Singapore

**Martin Preisig**

Switzerland

**George Prendergast**

USA

**Gareth Pryce**

UK

**Jude Przyborski**

Germany

**Carlo Pucillo**

Italy

**Chamindie Punyadeera**

Australia

**Lajos Pusztai**

USA

**Efisio Puxeddu**

Italy

**James Raftery**

UK

**Daniel Raftery**

USA

**Michael Ramharter**

Germany

**Padmanabhan Ramnarayan**

UK

**Nigel Rawson**

USA

**Prem-Veer Reddy**

USA

**Nancy Redeker**

USA

**Sally Redman**

Australia

**Andrew Rees**

Austria

**Juergen Rehm**

Canada

**Gregor Reid**

Canada

**Sirimon Reutrakul**

Thailand

**Giovanni Rezza**

Italy

**Anthony Rhodes**

Malaysia

**Andrew Rhodes**

UK

**Todd Richards**

USA

**Kristin Riekert**

USA

**Louise Robinson**

UK

**Kenneth Rockwood**

Canada

**Mauricio Rodrigues**

Brazil

**Fernando Rodríguez-Artalejo**

Spain

**Laura Rogers**

USA

**Monique Roobol**

Netherlands

**Ivan O. Rosas**

USA

**Amanda Ross**

Switzerland

**Jean Pierre Routy**

Canada

**Laurence Rubenstein**

USA

**Everardo Saad**

Brazil

**Caroline Sabin**

UK

**Amirhossein Sahebkar**

Iran

**Gil Salles**

Brazil

**Emilia Salvadori**

Italy

**Carole Samango-Sprouse**

USA

**Jeeva Sankar**

India

**Valéria Saraceni**

Brazil

**Jerome Sarris**

Australia

**Sivasankaran Sashidharan**

UK

**Naveed Sattar**

UK

**Robert Sauerwein**

Netherlands

**John Saxton**

UK

**Roberta Scherer**

USA

**Marjanka Schmidt**

Netherlands

**John Schneider**

USA

**Henrik Carl Schønheyder**

Denmark

**Mary Schooling**

Hong Kong

**Helmut Schröder**

Spain

**Marieke Schuurmans**

Netherlands

**Ian Scott**

Australia

**Paola Sebastiani**

USA

**Yoshihiko Seino**

Japan

**Lorenzo Sempere**

USA

**Jon Shaffer**

UK

**Dennis Shanks**

Australia

**Priyanka Sharma**

USA

**Neil Shear**

Canada

**Lauren Sherar**

UK

**Xiangyang Shi**

China

**Yufei Shi**

Saudi Arabia

**Kenji Shibuya**

Japan

**Aesun Shin**

Korea

**Steffen T Simon**

Germany

**Julie Simpson**

Australia

**Jasvinder Singh**

USA

**Anushua Sinha**

USA

**Melissa Southey**

Australia

**Martin Spahn**

Switzerland

**Joseph Sparano**

USA

**Claudia Spies**

Germany

**Robert Steele**

UK

**Benjamin Steinberg**

USA

**Karen Steindorf**

Germany

**Steven Steinhubl**

USA

**Andy Stergachis**

USA

**Rob Stockley**

UK

**Tim Stockwell**

Canada

**Trevor Stone**

UK

**Marie-Pierre St-Onge**

USA

**Sharon Straus**

Canada

**Elizabeth Streicher**

South Africa

**Karien Stronks**

Netherlands

**Rodney Stuck**

USA

**Erich Sturgis**

USA

**David Sullivan**

Australia

**Richard Sullivan**

UK

**David Sullivan**

USA

**Gerhard Sundborn**

New Zealand

**Ali Sunyaev**

Germany

**Mark Sussman**

USA

**Manikkam Suthanthiran**

USA

**Dallas Swendeman**

USA

**Jeffrey Swigris**

USA

**Matthew Sydes**

UK

**Ambrose Talisuna**

Kenya

**Shenglan Tang**

USA

**Alessandra Tavani**

Italy

**Antonio Teixeira**

Brazil

**Andrew Teodorczuk**

UK

**Gareth Thomas**

UK

**David Thomas**

USA

**Graham Thomas**

USA

**Mangesh Thorat**

UK

**Adam Timmis**

UK

**Francisco Tinahones**

Spain

**Richard Titball**

UK

**Nevins Todd**

USA

**Oyewale Tomori**

Nigeria

**Michela Torricelli**

Italy

**Carlos Tovilla-Zárate**

Mexico

**Jonel Trebicka**

Germany

**Kelton P Tremellen**

Australia

**François Treussart**

France

**Andrea Tricco**

Canada

**Irene Tuffrey-Wijne**

UK

**Sheila Turner**

UK

**Dirk Ubbink**

Netherlands

**John Urquhart**

USA

**Clarissa Valim**

USA

**Wim Van Den Heuvel**

Netherlands

**Jenny Van Der Steen**

Netherlands

**Tjip Van Der Werf**

Netherlands

**Daniëlle Van Der Windt**

UK

**Rogier Van Doorn**

Vietnam

**Pieter Van Gorp**

Netherlands

**David Van Wagoner**

USA

**Carlo Vancheri**

Italy

**Per Olav Vandvik**

Norway

**Per Vandvik**

Norway

**Lennert Veerman**

Australia

**Gerald Verhaegh**

Netherlands

**Sten Vermund**

USA

**Johny Verschakelen**

Belgium

**Laura Via**

USA

**Cecile Viboud**

USA

**Roderik Viergever**

USA

**Anne Vik**

Norway

**Joan Vilalta-Franch**

Spain

**Reina Villareal**

USA

**Jose Vina**

Spain

**Dietrich von Rosen**

Sweden

**Lorenz von Seidlein**

UK

**Edward Walker**

USA

**Lance Waller**

USA

**Jeremy Walston**

USA

**Eugene Haydn Walters**

Australia

**W Douglas Weaver**

USA

**Don Weiss**

USA

**Scott Weiss**

USA

**Christian Wejse**

Denmark

**Robert Welch**

USA

**Thomas Wellems**

USA

**Athol Wells**

UK

**Lynn Marie Westphal**

USA

**Ove Wiborg**

Denmark

**Paul Wicks**

USA

**Annelies Wilder-Smith**

Singapore

**Marko Wilke**

Germany

**Diana Wilkie**

USA

**Robert Wilkinson**

UK

**Karen Willard-Gallo**

Belgium

**Thomas Williams**

Kenya

**Christopher Williams**

UK

**Jennie A C Wilson**

UK

**Adam Witney**

UK

**Denise Wolf**

USA

**Paul Wolters**

USA

**Chansuda Wongsrichanalai**

Thailand

**Patrick CY Woo**

Hong Kong

**Junjie Xiao**

China

**Mingzhao Xing**

USA

**Y. Tony Yang**

USA

**Patsy Yates**

Australia

**Ding-Wei Ye**

China

**Bu Yeap**

Australia

**Neville Yeomans**

Australia

**Linwah Yip**

USA

**Joshua Yukich**

USA

**Basia Zaba**

UK

**Leo Zacharski**

USA

**Marco Zappa**

Italy

**Maximilian Zeyda**

Austria

**Yaobi Zhang**

Senegal

**George Zhang**

USA

**Qi Zhou**

Canada

**Jerry Zimmerman**

USA

**Armin Zittermann**

Germany

**Robert Zivadinov**

USA

**Bernard Zonnenberg**

Netherlands

**Manuel Zorzi**

Italy

**Maria Victoria Zunzunegui**

Canada

